# New Procedure for BIM Characterization of Architectural Models Manufactured Using Fused Deposition Modeling and Plastic Materials in 4.0 Advanced Construction Environments

**DOI:** 10.3390/polym12071498

**Published:** 2020-07-04

**Authors:** Daniel Diaz-Perete, Jorge Manuel Mercado-Colmenero, Jose Manuel Valderrama-Zafra, Cristina Martin-Doñate

**Affiliations:** Department of Engineering Graphics Design and Projects, University of Jaen, Campus Las Lagunillas, s/n. Building A3-210, 23071 Jaen, Spain; ddp00006@red.ujaen.es (D.D.-P.); jmercado@ujaen.es (J.M.M.-C.); jmzafra@ujaen.es (J.M.V.-Z.)

**Keywords:** FDM, plastic materials, polymers, topological optimization, construction 4.0, advanced digital geometries

## Abstract

This paper presents a new procedure for the building information modeling (BIM) characterization of structural topologies manufactured with plastic materials and fused deposition modeling (FDM) additive technology. The procedure presented here transforms the architectural geometry into an expanded three-dimensional model, capable of directly linking the topology of the plastic structure with the technological, functional and economic requirements for working in advanced construction 4.0 environments. The model incorporates a new algorithm whose objective is to recognize the topological surface of the plastic structural part obtaining in a fully automated way the FDM manufacturing time as well as the manufacturing cost. The new algorithm starts from the voxelized geometrical surface of the architectural model, calculating the manufacturing time from the full geometric path traveled by the extruder in a voxel, the extruder’s speed, the print pattern and the layer height. In this way it is possible to obtain a complete digital model capable of managing and analyzing the plastic architectural object in an advanced BIM 4.0 environment. The model presented in this paper was applied to two architectural structures designed for a real urban environment. The final structural geometries have been obtained through topological processes in order to reduce the raw plastic manufacturing material and to improve the plastic structure strength. The architectural elements have been validated structurally by the means of numerical simulations, following the scenario of loads and boundary conditions required for the real project. The displacement maps point to a maximum value of 0.5 mm according to the project requirements. The Von Mises stress fields indicate maximum values of 0.423 and 0.650 MPa, not exceeding in any case the tensile yield strength of the thermoplastic material.

## 1. Introduction

The use of polymeric plastic materials in the field of architectural construction has multiple advantages in terms of manufacturing and building free form surfaces. The design of plastic construction elements allows the recycling of materials, improving the architectural sustainability and the structural mechanical requirements.

Advanced technologies commonly used in the industrial manufacturing sector [1,2,3] are being progressively transferred to the field of architecture and construction. The construction sector has evolved slowly in recent years without achieving major digital changes. The particularities of the construction field constitute a challenge in the process of adapting technologies from the industrial area. Digital transformation in the construction sector, and in particular the potential of additive manufacturing technologies, could become a key technology for construction 4.0. There has been growing interest in additive processes for architectural and civil engineering over the past two decades [4]. The greatest challenge facing additive construction is the scaling-up of existing additive manufacturing technologies and the use of efficient and sustainable materials. Additive construction is largely dominated by the concrete–extrusion technology, like Gantry technologies [5], the Contour Crafting method [6,7,8], concrete printing [9,10], cable-suspended platforms [11,12] and the use of robots [13,14]. Despite the growing research on additive manufacturing [15,16,17,18] the developments and applications achieved to date in building applications are still in their initial stages [19].

Structural models take advantage of additive technology in developing complex shapes difficult to obtain using traditional manufacturing processes [20,21,22]. Topological optimization tools in conceptual stages allow for optimal geometries in accordance with the boundary conditions of the project. These methodologies help to determine in preliminary stages the desired process constraints, such as material properties, component geometrical features, loading conditions, and objective functions. The topological optimizer tool enhances the material distribution, presenting a structural layout that fulfills the aesthetic and functional specifications. The topological optimization process requires redesigning the original model by including a new complex structure. Topological optimization for buildings and other structural engineering functions has been used for research in several applications [23,24,25].

The design of architectural elements in 4.0 environments takes advantage of using building information modeling (BIM) methodologies, constituting one of the fundamental pillars in the management of the new construction industry 4.0. An adequate definition of BIM architectural objects adapted to new productions and materials can greatly help in the process of automating construction tasks, improving the efficiency and profitability of the process. Unfortunately, the current BIM management systems in 4.0 construction environments, and more specifically, using polymeric materials, are in their very initial stages. This situation prevents companies from responding with innovation to a sector that urgently needs the adaptation to customer´s requirements. 

Ensuring procedures that automate complex tasks, thus improving the management and execution of project activities, can greatly help to reduce development times. Similarly, the creation of digital platforms that incorporate tools and new procedures based on advanced design models could greatly help in collaborative tasks, improving communication and information exchange [26,27]. Building information modeling is a methodology that works with digital 4.0 platforms allowing collaborative work between different agents in construction projects [28,29]. The BIM methodology is implementable in digital environments, enabling working with complex digital models. Additionally, BIM methodology could manage all the project management information throughout the life cycle of the building [30]. According to the 2014/24/EU standard of the European Parliament, the use of BIM environments is mandatory in construction projects according to the requirements of each country.

Construction 4.0 environments require a treatment of three-dimensional models completely different from standard CAD design models, requiring expanded hybrid-type models that allow working with the geometry and the set of mechanical, chemical, elastic and other properties of the polymeric material simultaneously.

The BIM methodology is presented as a great advance, compared to the standard CAD design, expanding the possibilities of working with smart vertical applications specially designed for the smart resolution of particular problems. Digital modeling of architectural elements allows the exchange of information regarding physical and functional requirements and different software applications, as well as between members of a multidisciplinary team. BIM is in charge of managing the geometric and functional part specifications in the construction field [31,32,33,34]. However, there are still many problems and limitations in BIM platforms related to the uses of CAD information. Although BIM platforms allow working with CAD models, the proper mapping of geometric and non-geometric (functional) attributes for models designed to be built using additive manufacturing has not yet been thoroughly studied.

Materials are a very important part of 3D printing technology as they have to meet the stiffness and strength requirements of the additive manufacturing process. Polymers are highly appropriate for additive manufacturing in construction applications [35,36,37]. They present, as main features, low price and low density, as well as the possibility of storing them in controlled tanks unlike other materials such as cement. Large-scale construction processes are particularly vulnerable to the lack of accuracy, so in this way plastic materials have been considered appropriate for additive construction [36]. Recycled plastic materials are widely used in the additive manufacturing (AM) process. Waste plastic filament, misprints and undesired outputs can be reclaimed and reused. This could be an enabler and a driving force for improved construction sustainability [38]. Although in recent years the application of plastic materials in additive manufacturing had a conceptual character, at present fused deposition modeling (FDM) technology is evolving from rapid prototyping towards a rapid manufacturing method, changing the main purpose in producing finished components [39,40]. There are several approaches to the use of plastic materials in additive architectural construction. Following this line, Universe Architecture [41] developed a Landscape House made of a bio plastic compound of 80% vegetable oil and 20% concrete paste, and DUS Architects [42] printed an eight-square-meter house in Amsterdam using plastic materials. The principal motivation of these applications was to facilitate the construction of buildings that were eco-efficient in terms of their thermal, acoustic and structural properties, and which commonly incorporated potentially complex forms and geometries. Unfortunately, despite the advantages of the use of plastic materials in additive manufacturing for architectural purposes, they have been used in very few applications [43].

In order to resolve these issues, this paper presents an innovative procedure applicable to BIM structural objects made of plastic and additive FDM technology. The procedure presented transforms the geometry of the architectural object into an expanded three-dimensional digital model capable of directly linking the topological information of the structural element with the technological, functional and economic information necessary for its implementation in an advanced 4.0 construction environment. In this way, it is possible to obtain a complete digital model for structural elements made with polymeric materials and FDM technology. The model is capable of incorporating, automating and managing in real time the information and calculations necessary for manufacturing, validating and economically analyzing the plastic architectural element in an advanced BIM environment. The new model incorporates an innovative geometric algorithm whose objective is to obtain in a fully automated way the FDM manufacturing time for BIM structural plastic objects as well as the manufacturing costs. The new expanded digital model includes all the definition, properties and technological features required for the FDM manufacturing process, allowing the definition of any plastic structural element and new polymeric materials in an advanced BIM construction 4.0 environment, enhancing the advantages of using polymers in additive FDM construction environments.

## 2. Materials and Methods

### 2.1. Digital Reconstruction of the Architectural Environment

**Figure 1:**
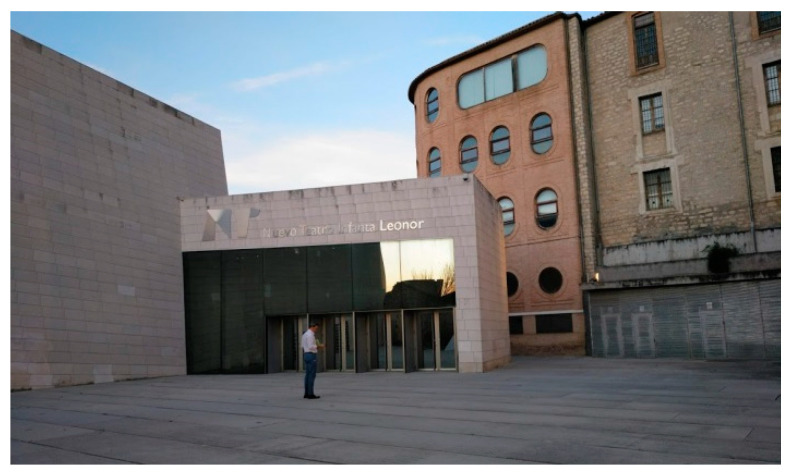
View of the theater [43] located on one side of the square.

**Figure 2:**
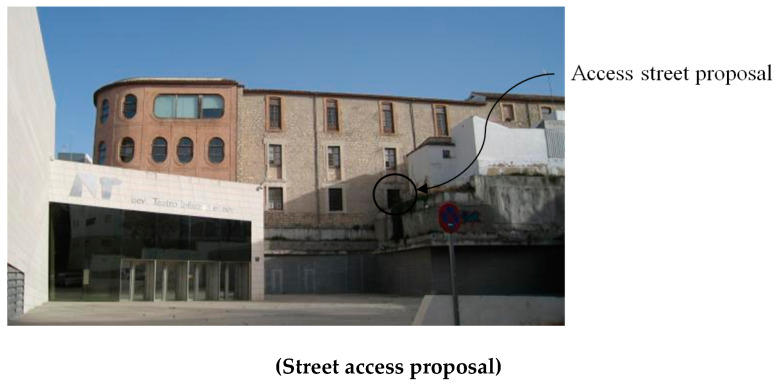
Details of the proposed access to the square from a road at the upper boundary, side view.

**Figure 3:**
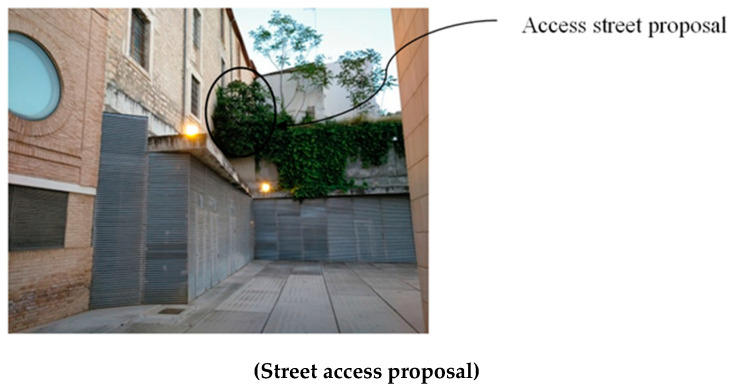
Details of the proposed access to the square from a road at the upper boundary, front view.

The architectural proposal presented in this manuscript is located in the urban environment of Jaen (Andalusia, Spain). In particular, this environment corresponds to a square in the old town, where a modern-style theater [44] with a minimalist façade is located (Figure 1). The square presents functional problems such as a lack of access from a road located at a higher level (Figure 2 and Figure 3). Additionally, the access to the square is limited to a single entrance of small dimensions, transforming the square´s function into a passage area (Figure 1).

To establish an architectural solution that gives additional access from the proposed route to the square a digital model was required of the architectural environment under study. The process of digital reconstruction consists of a prior analysis of the boundary conditions that surround the elements that have to be digitally modeled. This analysis involved an evaluation of the lighting level throughout the whole day, the geometrical and topological boundaries of the square, accessibility points, etc. In this way, it was possible to determine an appropriate and optimal process for data collection that could suit the specifications of the urban environment. For the initial study of the square’s requirements, the meteorological conditions of its location were taken into account, since they implied high levels of lighting intensity during most of the year. In addition, due to the material features presented in the façades and coatings of the square elements, the use of photogrammetric methods of the terrestrial field or close-up object with a vertical plane photography were established as the optimal process for collecting the digital data. These methodologies were characterized by the use of three-dimensional reconstruction techniques from the images obtained.

**Figure 4:**
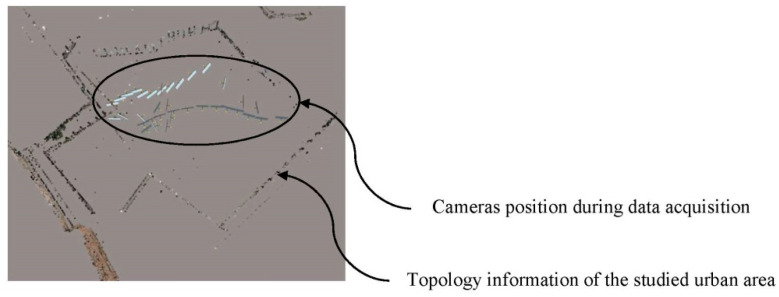
Arrangement of the cameras during the data collection of the urban environment.

The use of this methodology took advantage of using high levels of precision, in comparison with other conventional methods such as Google Earth [45] or Lidar data [46]. The images of the environment under study were taken with an OLIMPUS E-500 DSLR [47], with a 14–80 mm objective, and a fixed focal length of 30 mm. The shutter speed [47] was automatic including a value greater than 1/60 s in order to avoid photogrammetric measurement errors. Due to the geometrical and topological conditions of the urban environment, the data collection was determined following a previously defined optimized inner polygonal path (Figure 4). Furthermore, and in order to avoid a high light reflectance, the data collection process was carried out on a cloudy day, under homogeneous light conditions which eliminated the brightness.

**Figure 5:**
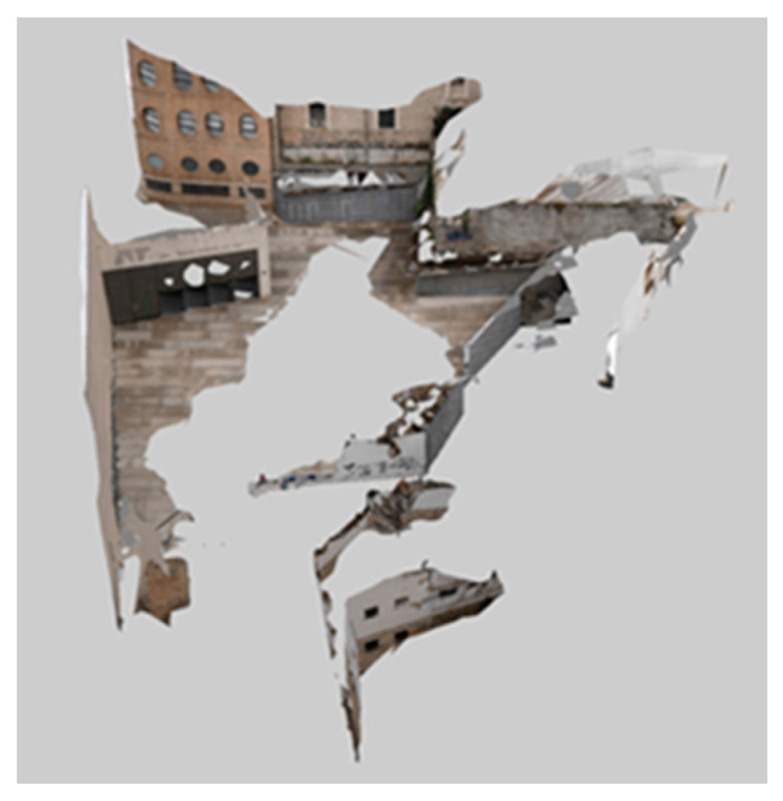
Digital model B’ obtained with terrestrial photogrammetry.

The photogrammetric information of the urban environment was processed with the ContextCapture software [48], using an orientation procedure for the photographs obtained. The software [48] uses an algorithm for matching and overlapping thousands of points acquired automatically in the images. For the digital square reconstruction, no information about its orientation or geolocation was required. That is, a relative coordinate system is defined. As shown in Table 1, the results of the automatic aerial triangulation process of the photographic data show a high level of precision for the image processing, establishing the discrete digital environmental model B’ (Figure 5).

Furthermore, 37 photographs were obtained in order to perform the digital model B’ and they were taken by an OLIMPUS E-500 DSLR [47] digital camera. Table 2 shows the technical characteristics and information related to each photograph taken and the digital camera used. On the other hand, the computational processing time used to obtain the digital model B’ was 4 min and 45 s. The average ground resolution was 0.0055 units per pixel and the ground coverage was 3841.44 square units. Likewise, the characteristics of the equipment used to perform the digital modeling were as follows: MSI notebook with a 2.80 GHz Intel (R) Core (TM) i-77700HQ CPU and the Nvidia Gforce GTX 10 series as a graphics card. 

The discrete three-dimensional model B’ resulting from the aerial triangulation was imported for a manual post-processing into the commercial Bentley Microstation software [49]. This process requires an orientation and scaling of the primitive discrete model B’. For this, a set of linear measurements M_i_ ∀ i [1, n] were determined with n being the number of measurements in the urban environment, obtained with a Leica Disto D510 (E7500i) digital laser distance meter [50]. Table 3 shows the technical specifications for this measuring device. Additionally, the process of taking linear measurements M_i_ was completed using GPS coordinates Gj ∀ j [1, m], m being the number of coordinates in the ETRS89-UTM system. In this way, from the geometrical operations of plane translation, rotation and scale, the discrete model B’ is established (Figure 5). On the other hand, for the geographic positioning process of the discrete mesh B’, a centimeter precision Trimble Geoexplorer 7X [51] GPS device was used.

**Figure 6:**
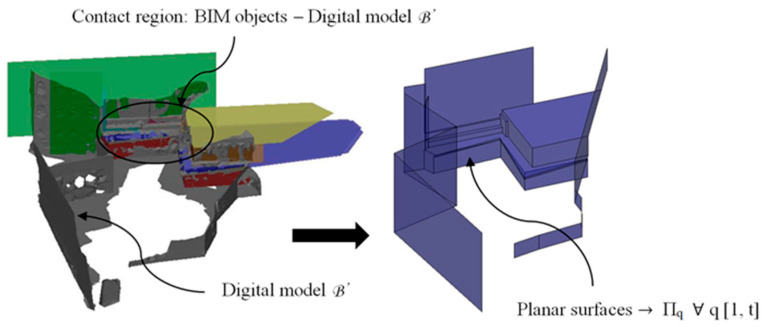
Π_q_ planes in the support areas of the building information modeling (BIM) objects and the final result of the digital reconstruction of the environment.

The constructive and structural solutions that have been proposed to solve the architectural problems exposed in this manuscript are defined digitally using BIM-type objects. These are therefore compatible with the discrete model B’ of the urban environment obtained (Figure 5). Therefore, in order to obtain a maximal precision, in those areas where BIM objects will be located, the data of the mesh B’ are validated with the dimensional information obtained with a Leica Disto D510 (E7500i) digital laser [50]. To ensure a high precision in the contact areas as well as compatibility between the BIM objects and the discrete model B’, a set of flat surfaces Π_q_ ∀ q [1, t] were modeled, with t being the number of flat surfaces. Figure 6 shows the contact areas between B’ and the BIM objects as well as a detailed design of the flat surfaces Π_q_ and the final result of the digital reconstruction of the environment.

### 2.2. Structural Design of Constructive BIM Elements

**Figure 7:**
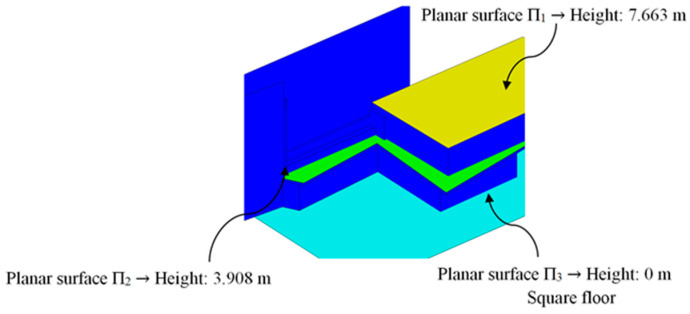
Definition of the flat surfaces that define the geometric boundary conditions of the BIM construction elements.

The main function of the proposed BIM constructive objects was to provide an additional access (Figure 2 and Figure 3) from a pedestrian path located at a higher elevation, currently unused. In this way, linearity in traffic and communication between the north and southwest ends of the square is favored. The design of the proposed BIM construction elements was carried out based on the set of geometrical, topological and functional environmental requirements as well as the technical specifications required for the CTE Spanish technical building code [52]. The new proposed BIM construction elements were defined as a set of stairs designed to be manufactured using fused deposition modeling (FDM) technology. Given the topology of the urban environment and the difference in elevation between the access of the proposed pedestrian path and the square under study, it is proposed to divide the BIM construction element into two different elements [E_1_ E_2_]. In this way, this proposal optimizes the useful volume of the urban environment, favoring its visual impact and the pedestrian traffic. The first BIM construction element, E_1_, provides access from the proposed pedestrian path (Figure 2 and Figure 3) to a Π_2_ plane located at a height of 3.908 m (Figure 7); while the second element, E_2_, located at a height of 3.908 m, guarantees the continuity of the designed BIM elements [E_1_, E_2_] between plane Π_2_ and planar surface Π_3_ at height 0 m of the square floor under study (Figure 7).

**Figure 8:**
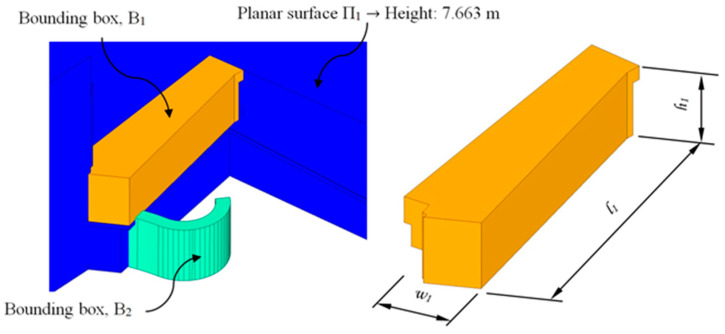
Generation of the B_1_ bounding box of the BIM construction element E_1_ based on the geometric and topological requirements of the urban environment.

Firstly, in order to determine the geometrical design of the construction element E_1_ corresponding to the first section (determined by the flat surfaces Π_1_ and Π_2_, Figure 7), a control volume (Figure 8) or bounding box B_1_ of the maximal dimensions l _1_, w_1_ and h_1_ was defined. As shown in Figure 8, the geometry of the bounding box was adapted to the topological requirements of the urban environment. In order to do this, its geometrical modeling was carried out using a set of subtraction-type Boolean operations, establishing the plane surfaces Π_q_ (Figure 6) of the urban environment as a reference geometry. In this way, it was possible to generate a control volume B_1_ for modeling the final geometry of the constructive element E_1_.

**Figure 9:**
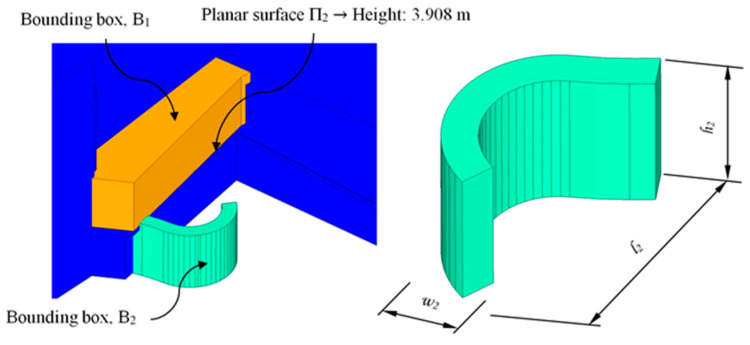
Generation of the B_2_ bounding box of the BIM E_2_ construction element based on the geometric and topological requirements of the urban environment.

Similarly, for section E_2_ of the BIM construction element, a second bounding box B_2_ with the maximum dimensions l _2_, w_2_ and h_2_, was defined. B_2_ was adapted to the geometry and topology of the urban environment from the flat surface Π_2_. As shown in Figure 9, its design provided continuity between the flat surfaces Π_2_ and Π_3_, while its radial geometry optimized the useful traffic volume of the square under study. Table 4 shows the maximum dimensions of each previously defined bounding box, B_1_ and B_2_.

**Figure 10:**
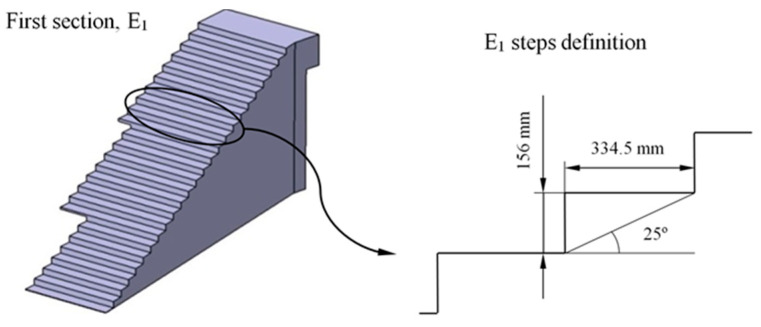
Definition of the steps for the first section of the E_1_ construction element.

**Figure 11:**
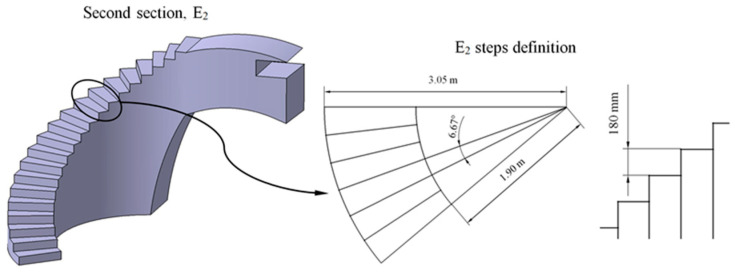
Definition of the steps for the second section of the E_2_ construction element.

BIM elements E_1_ and E_2_ are designed from the generated control volumes B_1_ and B_2_ (Figure 8 and Figure 9). The dimensions relative to the footprint and counter-footprint of each step for both elements E_1_ and E_2_ were established according to the requirements of the Technical Building Code [52] for stairs and ramps in public spaces. The first part E_1_ of the construction element presents a drop of 3633 m. Therefore, as shown in Figure 10, 24 steps were defined with a 334.5 mm footprint, a 156 mm counter-footprint with an angle of 25°. The second section E_2_ of the construction element presents a difference of 3.908 m. Therefore, as shown in Figure 11, 20 steps were defined with a counter-tread size of 180 mm and an angle of 6.67°. In a similar way, the inner radius of this section was 1900 m and the outer radius 3050 m. On the other hand, the BIM elements were subjected to the corresponding surface treatments to fulfil the requirements established in the Technical Building Code [52] for their correct implementation in the urban environment.

**Figure 12:**
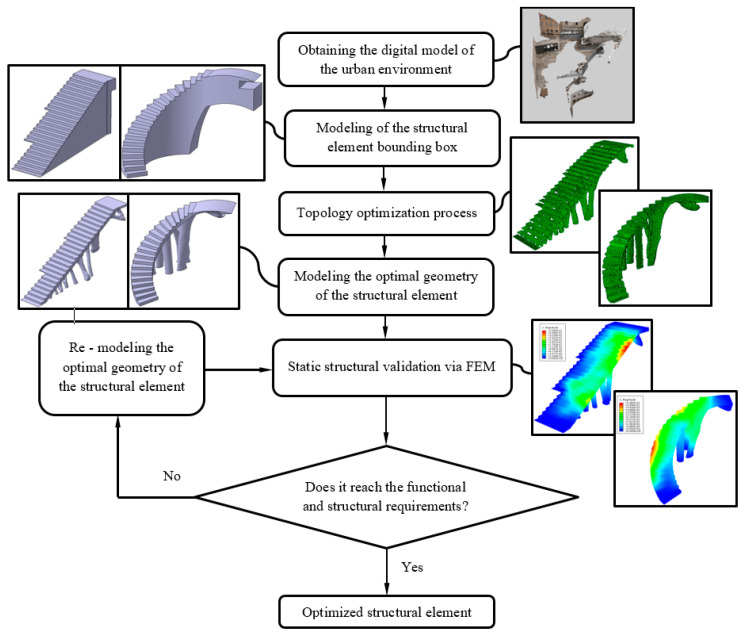
Flow diagram for the methodology used in the geometric and structural design of the BIM construction elements under study.

The design of the steps, in the upper areas of the BIM construction elements, was carried out using Catia V5 parametric software [53], starting from the volumes B_1_ and B_2_ (Figure 8 and Figure 9) generated previously in Bentley Microstation [49] software. Volumes B_1_ and B_2_ were adapted using Boolean subtraction operations. Then, the designed elements E_1_ and E_2_ (Figure 10 and Figure 11) were used as a reference for the definition of the optimized final geometry of the construction element with plastic materials. Figure 12 shows and summarizes, through a flow diagram, the methodology developed including the different processes and operations used to define the final CAD geometry of the BIM construction elements under study.

#### 2.2.1. Definition of the Plastic Material for the Additive Manufacturing of BIM Elements

The manufacture of the BIM elements defined in this manuscript was carried out using polyethylene terephthalate glycol (PETG) plastic material. This polymeric material is a derivative of polyethylene terephthalate (PET), produced through a copolymerization process. This polymeric material is widely used in the additive manufacturing process due to its excellent qualities to be thermoformed and extruded. On the other hand, this plastic material has good thermal stability, low moisture absorption, non-slip surface finishes, greater wear resistance, resistance to gamma radiation, resistance to corrosion against oxidizing agents and is recyclable. These features make the PETG plastic material ideal for designing the parts and elements located outside where they are subject to high temperature gradients produced during daily cycles. For instance, this polymer has previously been used in architectural elements such as skylights, walls, handrails or as a substitute for glass in bus shelters. After the manufacturing process, this polymeric material presents good cohesion between the layers, showing optimal mechanical capabilities under unidirectional tensile and compressive load states. In addition, it has a high resistance to impacts and great wear strength.

In terms of manufacturing, it is a versatile and easy-to-print material, its extrusion temperature is 235 °C, with 70 °C being the temperature of the 3D printer bed. During its manufacturing process, the viscosity of the melting front presents high adhesion between the adjacent layers. Similarly, the manufacturing parameters of the plastic material (see Table 5) reduce the defectology associated with cases of excessive viscosity, such as the lifting of previous layers which involves the generation of stress concentration between layers. Table 5 shows the magnitude of each of the physical, mechanical and technological properties of the PETG used for the design of the construction elements defined in this manuscript.

#### 2.2.2. Application of Topological Optimization Algorithm for the Design of Plastic BIM Structural Elements

To complete the geometrical definition of the BIM elements under study, it was proposed to use topological optimization algorithms on the previously defined bounding boxes B_1_ and B_2_ (Figure 10 and Figure 11). The application of the topological optimization algorithms was carried out using the numerical and commercial software FEM Abaqus CAE [54], in particular the TOSCA [55] calculation engine. The use of these algorithms optimized the geometry and the stiffness–mass relation of the plastic BIM elements under study. Based on the definition of the static structural numerical analysis, which represented the state of loads and boundary conditions to which the construction elements were subjected, the topological optimization algorithm proposed an optimized final design adapted to this analysis. In other words, this optimization process determines the geometrical areas of the bounding boxes B_1_ and B_2_ (Figure 10 and Figure 11) that ensure the structural integrity of the building elements and the areas that can be eliminated in order to improve structural efficiency. Generally, the geometries resulting from such optimization algorithms present difficulties in manufacture with conventional manufacturing techniques. However, given the versatility of the additive manufacturing process, both BIM plastic construction elements could be implemented in the urban environment following the geometry that resulted from the topological optimization algorithm. 

**Figure 13:**
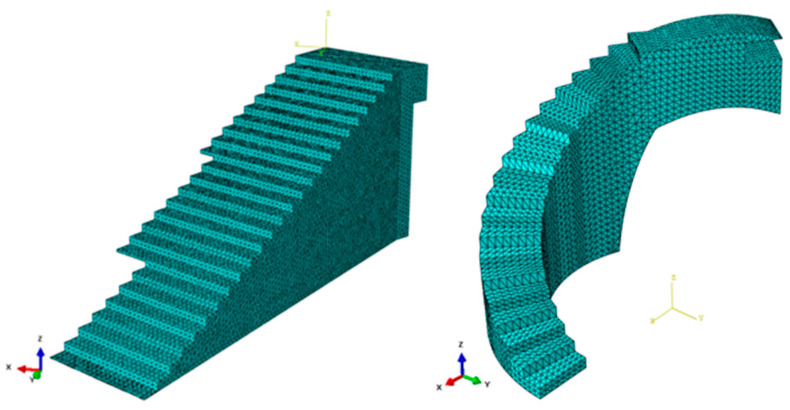
Discrete mesh associated with the bounding boxes B_1_ and B_2_.

**Figure 14:**
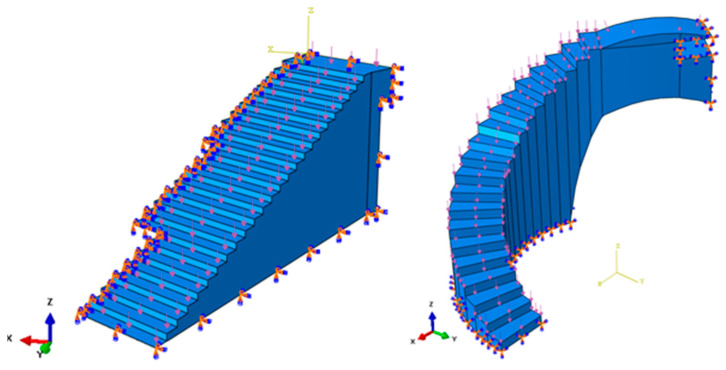
Load scenario and the boundary conditions associated with the bounding boxes B_1_ (**left**) and B_2_ (**right**).

According to the topologies of the construction elements under study and the typology of the structural numerical analysis, the bounding boxes B_1_ and B_2_ were discretized from three-dimensional tetrahedron-type elements (Figure 13 and Figure 14). In this way, during the topological optimization process, each tetrahedral element was associated with a control variable that took the value 1 if the element belonged to the optimal geometry, or otherwise, 0. Formally, the topological optimization problem is defined from the objective functions and the constraints of each variable that intervenes in the optimization problem (Equation (1)):

(1) $$\begin{array}{c} \min:\\ x \end{array}\;\begin{array}{c} f\left(x\right) \end{array}\;\;\;\mathrm{subject}\;\mathrm{to}:\;x\in D,\;x\in {\left\{0,1\right\}}^{n}$$

where D ⊃ ℝ^n^ represents the variable constraints of the optimization problem and f(x) represents the objective function to be optimized. To complete the optimization process of the structural element under study, the SIMP method [56] was implemented. This consists of replacing the objective function f(x) with a function of the type f(x^p^) (Equation (2)):

(2) $$x^{p}=\left\{x_{e}^{p},\;\ldots ,{\;x}_{e}^{p}\right\},\;|\;p>0$$

As well as the constraint function x ∈ {0, 1}^n^ by x ∈ [0, 1]^n^, the optimization problem applying the SIMP method takes the following form (Equation (3)):

(3) $$\begin{array}{c} \min:\\ x \end{array}\;\begin{array}{c} f\left(x^{p}\right) \end{array}\;\;\;\mathrm{subject}\;\mathrm{to}:\;x\in D,\;x\in {\left[0,1\right]}^{n}$$

The purpose of the case studies presented is to optimize the structural efficiency of the BIM plastic construction elements. Therefore, the objective function established for the topological optimization algorithm minimizes the deformation energy. The main constraint, to which the objective function is subject, is to limit the volume of bounding boxes, associated to the BIM construction elements. These volumes have been constrained to under 10% of the original volume for bounding box B_1_ (Figure 10) and under 20% of the original volume for bounding box B_2_ (Figure 11), generating the optimized geometries that satisfy the structural and functional requirements. Stiffness is the qualitative parameter for measuring strength against material elastic strains. This parameter presents the capacity of the structural element to withstand stresses without acquiring large strains. By including the study variables discussed above, it is possible to obtain the main equations that define the topological optimization problem (Equation (4)).

$$\begin{array}{c} \min:\\ x \end{array}\;\begin{array}{c} f\left(x^{P}\right)=F^{T}\cdot U=U^{T}\cdot K\cdot U \end{array}$$

$$D\left(x\right)=\;\rho {\left(x\right)}^{p}\cdot D^{0}\;|\;p>1$$

(4) $$\int_{V}\rho \left(x\right)\cdot \mathrm{dV}\leq \mathrm{VF}\;|0<\rho_{\min}\leq \rho \left(x\right)\leq 1,\;x\;\in V\;$$

where ρ(x) represents the density of the structural element under study, D_0_ represents the elastic properties of the isotropic material and ρ represents a penalty parameter. It was necessary to determine the density function of the construction elements since their volume was defined as the integral of the density function by the volume differential (Equation (5)). Density was interpolated between the material properties 0 and D_0_ (Equation (5)). It should be noted that a lower density limit $\rho_{\min}$ was introduced to avoid any type of singularity in the equilibrium problem. Generally, this lower density limit is equal to 10^−3^:

(5) $$D\;\left(\rho =0\right)=0,\;D\left(\rho =1\right)=D^{0}\;$$

For the strain energy minimization problem, there are two optimal solutions (ρ = 0, ρ = 1) if a penalty parameter ρ ≤ 3 is used. The topological optimization problem associated with minimizing the strain energy of the construction elements is presented in Equation (6). This function is subject to the constraints defined from its density function:

$$\begin{array}{cc} \begin{array}{c} \min\\ u\in U,\rho \end{array} & F^{T}U \end{array}$$

$$\mathrm{subject}\;\mathrm{to}:K\left(D\right)\cdot U=F\to D\left(x\right)=\rho {\left(x\right)}^{p}\cdot D^{0},$$

(6) $$\int_{V}\rho \left(x\right)\cdot \mathrm{dV}\leq \mathrm{VF}\;;0<\rho_{\min}\leq \rho \leq 1\;$$

In this way, starting from the initial geometries, a final geometry optimized in terms of structural efficiency was obtained. The new geometry was lighter, meeting the requirements of the load scenario and boundary conditions without significantly reducing the stiffness of the assembly. Thus, the geometries of the construction elements that maximized the structural efficiency in their designs were obtained.

The numerical simulations carried out for the optimization of the structural element assumed that the numerical models of numerical calculation were linear and static. The initial geometries of bounding boxes B_1_ and B_2_ (Figure 10 and Figure 11) were discretized by the means of solid structural tetrahedral elements of the C3D10 type. These elements with quadratic displacement behavior were made up of 10 nodes (four nodes at the vertices of the tetrahedron and six at the midpoints of its edges), having three degrees of freedom per node, including the translation in the X, Y and Z directions. In order to define the discrete meshes, a sizing operation with a magnitude of 100 mm for bounding box B_1_ and 120 mm for bounding box B_2_ (Figure 13) was used. This operation determines the approximate average size of each mesh element. Figure 13 show the meshes used in the topological optimization algorithm. Table 6 shows the statistics of the meshes used in the topological optimization algorithm.

The plastic material used to define the construction elements was PETG. In order to implement the thermoplastic material in the topological optimization algorithm, the magnitudes of the elastic properties of the material, Young’s modulus and Poisson’s ratio, as well as the magnitude of the density variable must be established. Table 6 shows the magnitudes of the variables used in the definition of the geometrical optimization of the structural element. It should be noted that the material is defined as elastic and isotropic, since the optimization process was carried out under the elastic regime of the material [39]. In a similar manner, given the modeling of the PETG, the Large displacement option was activated in the definition of the Solver to ensure that the final solution of the numerical model converged.

Figure 14 shows the load scenario and the boundary conditions to which the bounding boxes B_1_ and B_2_ were associated to with the construction elements under study. The load scenario was made up of a set of forces applied to each of the steps that made up the construction elements. The direction of the force was normalized to its application surface (Figure 14). According to the Spanish CTE technical building code [52], the magnitude of each force on each step uniformly applied has to be 10,000 N. On the other hand, according to the fasteners that the construction elements presented in the urban environment, embedment boundary conditions were defined on the surfaces that will be in contact with the support elements (Figure 14). The design area of the model that will be modified during the optimization and the freezing area or regions that were excluded from the optimization process were also defined. The design area was the whole domain of the construction elements and the freezing area of the set of surfaces where the forces will remain unchanged during the application of the topological optimization algorithm. In this way, it was possible to avoid excessive material removal and maintain the functional objective of the steps of the construction elements.

**Figure 15:**
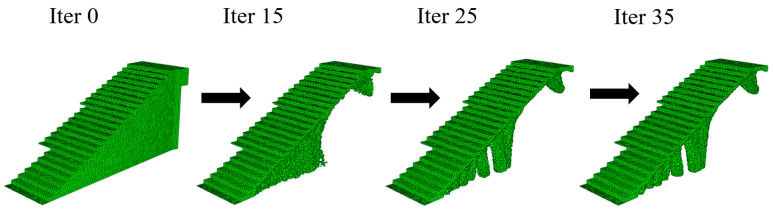
Evolution of the geometry of the bounding box B_1_ throughout the topological optimization process.

**Figure 16:**
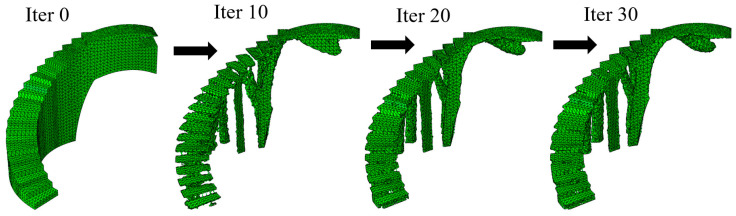
Evolution of the geometry of the bounding box B_2_ throughout the topological optimization process.

**Figure 17:**
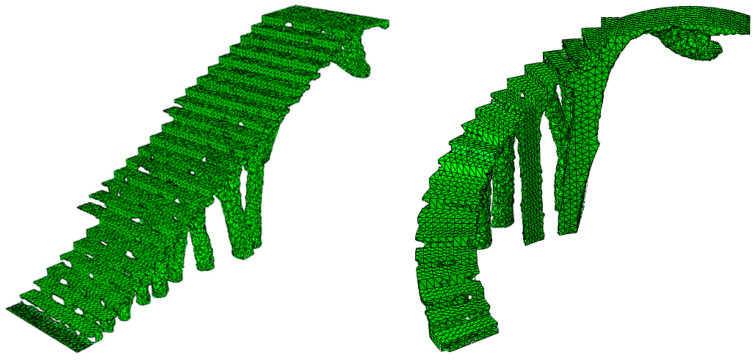
Final result of the optimization algorithm for the bounding boxes B_1_ (**left**) and B_2_ (**right**).

After 35 and 30 iteration cycles of the topological optimization algorithm, the final result of the optimal geometries is presented. Figure 15 and Figure 16 show the evolution of the geometries during the topological optimization process. It is possible to monitor, iteration after iteration, the volume reduction of the bounding boxes associated with the construction elements under study and how this is carried out maintaining the optimal structural efficiency which allows the functional requirements to be met under the load scenario to which these elements are submitted (Figure 17).

Table 7 shows the magnitude of the resulting volumes associated with the bounding boxes B_1_ and B_2_ (Figure 17), as well as the percentage of reduced volume obtained.

#### 2.2.3. CAD Geometrical Modeling of BIM Construction Elements

Starting from the discrete results of the topological optimization algorithm, the final geometrical modeling of the BIM construction elements was carried out. This new modeling process was based on the geometrical operations defined in a virtual CAD environment. The objective was to generate smooth and tangentially adjusted surfaces so that they can be adapted to the geometry of the discrete models generated from the topological optimization algorithm. The commercial software used to carry out this CAD modeling process was CATIA V5-R21 [53]. 

**Figure 18:**
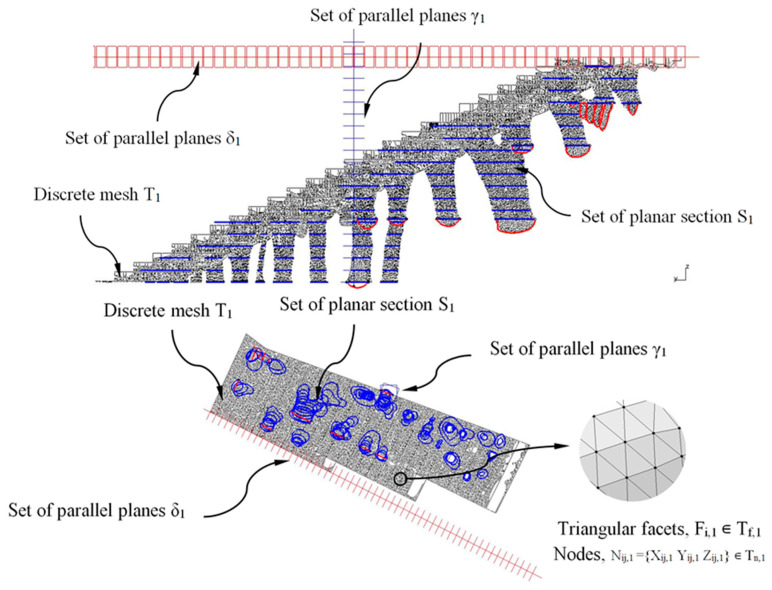
Definition of the sections S_1_ generated by the Boolean operation of the intersection between the discrete mesh T_1_ and the set of planes δ_1_ and γ_1_.

**Figure 19:**
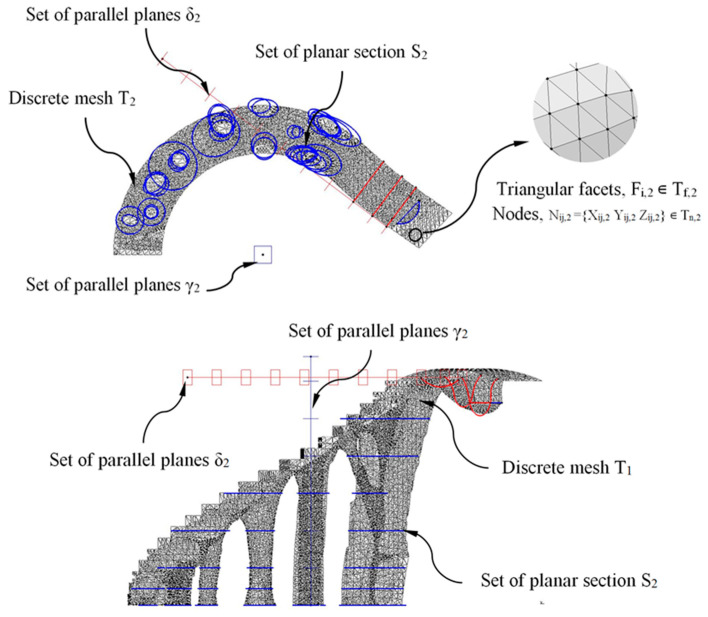
Definition of the sections S_2_ generated by the Boolean operation of intersection between the discrete mesh T_2_ and the set of planes δ_2_ and γ_2_.

The methodology for obtaining the final CAD modeling of the construction elements was based on intersecting the discrete three-dimensional meshes resulting from the topological optimization algorithm with a set of parallel planes, thus obtaining a new set of horizontal sections (Figure 18 and Figure 19). The set of parallel planes was established according to the direction of their normal vectors and with constant separation. The separation between each defined plane for the constructive element E_1_ is constant and equal to 200 mm, and for the constructive element E_2_ is 600 mm. The selection of the distance between the planes is established to ensure that from the resulting sections (Figure 18 and Figure 19) the CAD geometry of each construction element can be defined, adjusted, homogenized and optimized in order to capture the geometric details relative to the meshes resulting from topological optimization. As shown in Figure 18 and Figure 19, the discrete three-dimensional meshes, determined as T_1_ and T_2_, were composed of a set of triangular facets F_i,1_ ∊ T_f,1_ and F_i,2_ ∊ T_f,2_; and a set of nodes N_ij,1_ = {X_ij,1_ Y_ij,1_ Z_ij,1_} ∊ T_n,1_ and N_ij,2_ = {X_ij,2_ Y_ij,2_ Z_ij,2_} ∊ T_n,2_ being T_f,1_ and T_f,2_ arrays with rank n_1_x_3_ and n_2_x_3_; and T_n,1_ and T_n,2_ arrays with rank 3·n_1_x_3_ and 3·n_2_x_3_ where n_1_ and n_2_ represent the total number of triangular facets F_i,1_, F_i,2_ in E_1_ and E_2_ (Equation (7)). The set of parallel planes γ_1_, γ_2_ (for the discrete meshes T_1_, T_2_ associated with the structural elements E_1_, E_2_) have as a normal vector the Z axis direction according to the coordinate system (Figure 18 and Figure 19):

$$\begin{array}{cc} T_{1} & T_{2} \end{array}\in M_{1x2}\;\left(\mathbb{R}\right)\to \begin{array}{cc} T_{1}=\left\{T_{f,1}{\;T}_{n,1}\right\} & T_{2}=\left\{T_{f,2}{\;T}_{n,2}\right\} \end{array}$$

(7) $$\begin{array}{cc} T_{f,1}\in \;M_{n_{1}\mathrm{xm}}\;\left({\mathbb{R}}^{3}\right) & T_{f,2}\in \;M_{n_{2}\mathrm{xm}}\;\left({\mathbb{R}}^{3}\right)\; \end{array};\begin{array}{cc} T_{n,1}\in \;M_{3\cdot n_{1}\mathrm{xm}}\;\left({\mathbb{R}}^{3}\right) & T_{n,2}\in \;M_{3\cdot n_{2}\mathrm{xm}}\;\left({\mathbb{R}}^{3}\right) \end{array}\;$$

To improve the CAD model process, a second set of parallel planes δ_1_, δ_2_ for the meshes T_1_ and T_2_ with a normal vector orthogonal to the Z axis direction was generated. In Figure 18 and Figure 19, the planes γ_1_, γ_2_ and δ_1_, δ_2_ were represented in blue and red, respectively_._ As shown in Figure 18 and Figure 19, the set of flat sections S_1_ and S_2_ located in each discrete mesh under study were obtained by applying a Boolean operation of intersection between the set of parallel planes δ_1_, γ_1_, and δ_2_, γ_2_ and the discrete meshes T_1_ and T_2_ (Equation (8)). 

It should be noted that when operating on discreet meshes with triangular facets, the different sections S_1_ and S_2_ have to be smoothed and rounded in order to avoid generating stress concentrators in the final geometry for the construction elements. This fact also improves the aesthetics of the final design, surface finishing and manufacture:

$$\forall \;\left[\begin{array}{cc} \delta_{1} & \gamma_{1} \end{array}\right]\;\exists {\;S}_{1}\;|\;S_{1}=T_{1}\;\cap \;\left[\begin{array}{cc} \delta_{1} & \gamma_{1} \end{array}\right]$$

(8) $$\forall \;\left[\begin{array}{cc} \delta_{2} & \gamma_{2} \end{array}\right]\;\exists {\;S}_{2}\;|\;S_{2}=T_{2}\;\cap \;\left[\begin{array}{cc} \delta_{2} & \gamma_{2} \end{array}\right]$$

**Figure 20:**
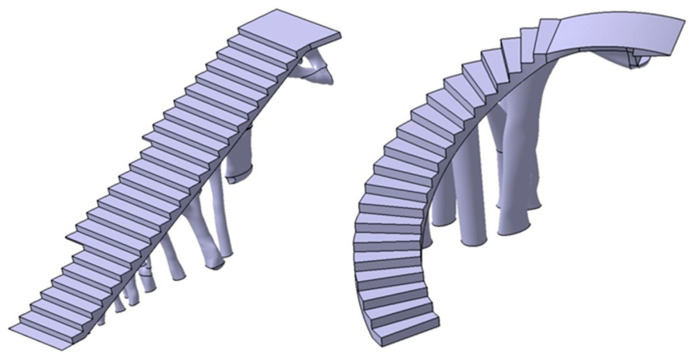
Final CAD model of the BIM type construction elements, E_1_ (**left**) and E_2_ (**right**).

After obtaining the geometry of the sections S_1_ and S_2_ and maintaining the commercial software CATIA V5-R21 [53] as the geometric CAD environment, the final CAD geometry of elements E_1_ and E_2_ were defined (Figure 20). To do this, a modeling multisection procedure was used, for those regions where elements present curvature changes in their geometry, sweep modeling for those regions that present a constant section throughout their spine; pad operations, to define a homogeneous geometry of the steps; and edge fillet, to round those areas that do not present points of tangency to each other. As can be seen (Figure 20), the final CAD geometrical design of the BIM plastic construction elements have very complex geometries formed by free-form surfaces. 

The manufacture of this type of geometry poses a lot of difficulties when using conventional manufacturing techniques. However, with the recent technological development of additive manufacturing techniques, the manufacturing of the construction elements under study can be carried out using FDM 3D printers and polymeric materials.

Once the final CAD models of each BIM construction element were defined, the resulting geometry was to be validated from static structural numerical analysis. That is, from the scenario of loads and boundary conditions previously defined for the topological optimization process (Figure 14), the stress map and the strain field obtained for the structural elements E_1_ and E_2_ were evaluated. In this way, it was possible to verify that the final CAD geometry of the elements E_1_ and E_2_ met the mechanical and structural requirements that the Spanish CTE technical building code [52] established for this type of element. 

#### 2.2.4. Numerical Structural Analysis of the CAD Geometry of BIM Plastic Construction Elements

**Figure 21:**
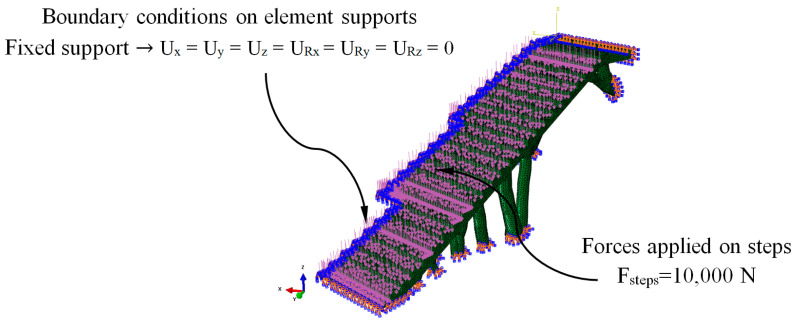
Load scenario and the boundary conditions of the numerical simulations for E_1_.

**Figure 22:**
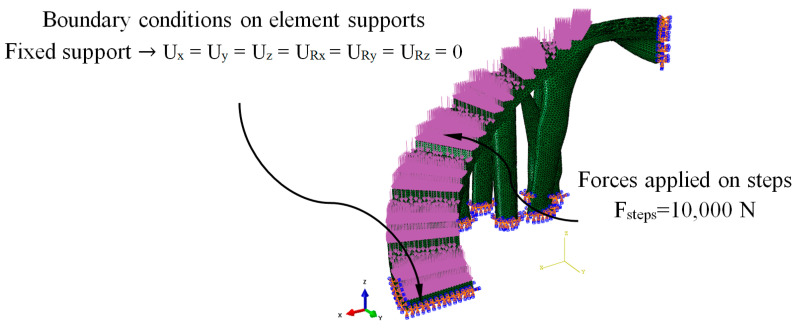
Load scenario and the boundary conditions of the numerical simulations for E_2_.

The numerical FEM software used to perform the numerical simulations of the mechanical and structural behavior of the BIM construction elements under study was Abaqus CAE [54]. Figure 21 and Figure 22 show the load scenario and the boundary conditions to which the construction elements under study were subjected (Figure 20). As it is shown, the load scenario and the boundary conditions were analogous with those previously used to define the topological optimization process of the geometries under study (Figure 14).

The plastic material used to define the construction elements under study was PETG. To implement the thermoplastic material in the static numerical analysis, the magnitudes of the material elastic properties of Young’s Modulus and Poisson’s coefficient were established. Table 5 shows the magnitudes of the variables used in the definition of the static structural numerical analysis. It should be noted that the material was defined as elastic and isotropic, since the mechanical behavior of the elements under study develops under an elastic, static and linear regime of the material [39], based on the load scenario and the boundary conditions presented in Figure 21 and Figure 22.

**Figure 23:**
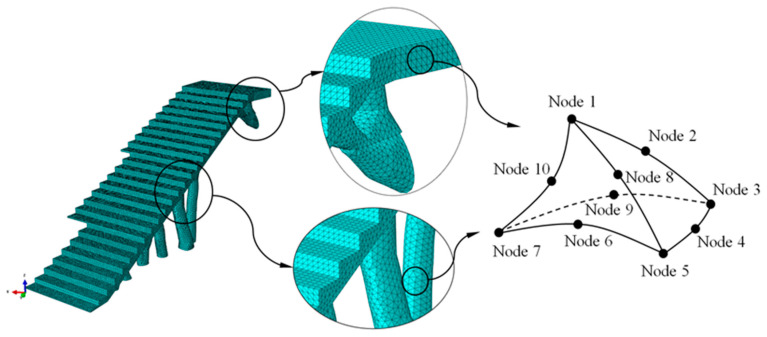
Mesh generated for the mechanical simulation, the BIM building element E_1_.

**Figure 24:**
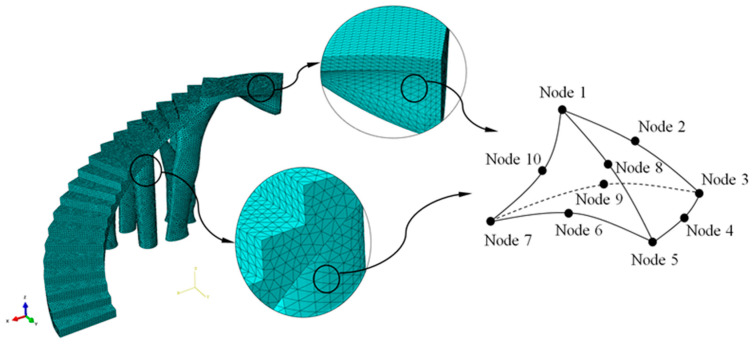
Mesh generated for the mechanical simulation, the BIM building element E_2_.

On the other hand, the geometries of the constructive elements E_1_ and E_2_ (Figure 20) are discretized, by means of solid structural tetrahedral elements of the C3D10 type (Figure 23 and Figure 24). These elements have a quadratic displacement behavior, are made up of 10 nodes (four nodes at the vertices of the tetrahedron and six at the midpoints of the edges of the tetrahedron) and have three degrees of freedom at each node: translation in the nodal X, Y and Z directions. To define the described meshes of the BIM E_1_ and E_2_ construction elements (Figure 20), the sizing operations were used to establish the average size of the tetrahedral elements (Figure 23 and Figure 24). For the first construction element E_1_, the magnification of the sizing was 60 mm and for the second construction element E_2_, the magnification of the sizing was 50 mm. Figure 23 and Figure 24 show the meshes used for linear and static mechanical simulations, and Table 8 shows the statistics of the meshes used for the linear and static mechanical simulations.

**Figure 25:**
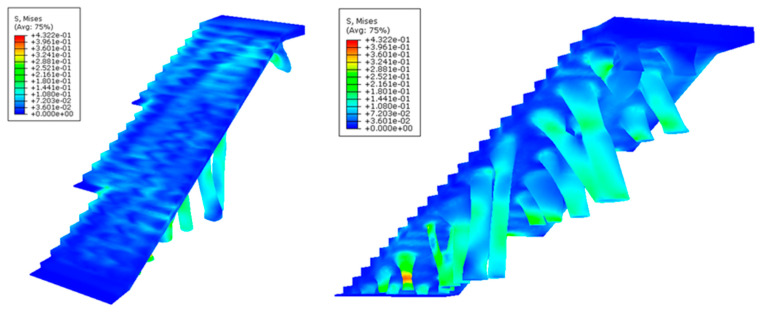
Field of the von Mises stress (MPa) obtained in the mechanical simulations: the BIM building element E_1_ indicated in two perspectives in the left and right pictures.

**Figure 26:**
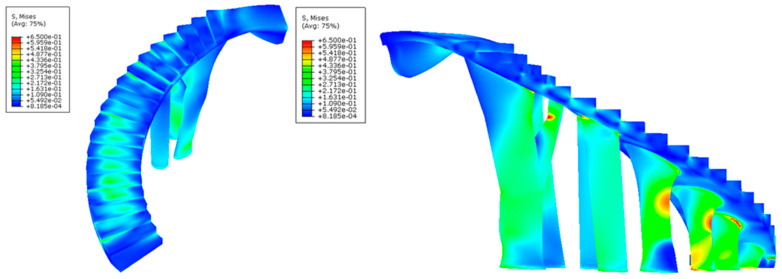
Field of the von Mises stress (MPa) obtained in the mechanical simulations: the BIM building element E_2_ indicated in two perspectives in the left and right pictures.

**Figure 27:**
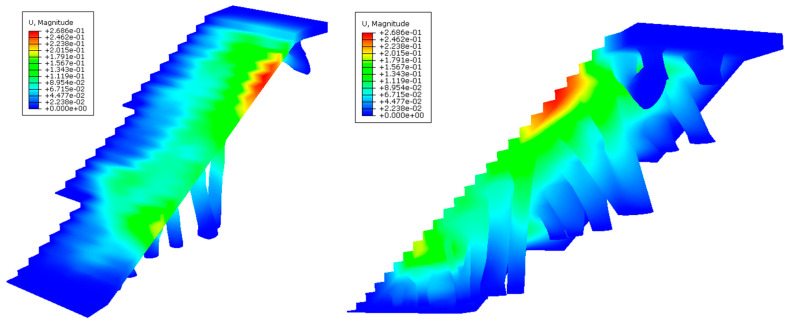
Field of displacements (mm) obtained in the mechanical simulations: the BIM building element E_1_ indicated in two perspectives in the left and right pictures.

**Figure 28:**
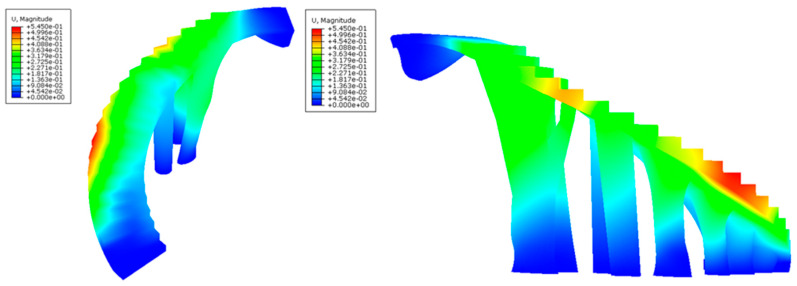
Field of displacements (mm) obtained in the mechanical simulations: the BIM building element E_2_ indicated in two perspectives in the left and right pictures.

After defining the set of pre-processing operations for the numerical simulations of the BIM construction elements, Figure 25, Figure 26, Figure 27 and Figure 28 and Table 9 present the results obtained from the displacement field and the von Mises stress field throughout the geometries analyzed.

As shown in Figure 25, Figure 26, Figure 27 and Figure 28 and Table 9, the maximum value in the von Mises stress field for the BIM construction elements under study is 0.423 MPa (E_1_, Figure 25) and 0.650 MPa (E_2_, Figure 27, respectively. According to Table 5, the tensile yield strength of the thermoplastic material used for the geometries under study is 50 MPa. In this way, it is verified that the thermoplastic material behaves in an elastic manner and does not suffer in any region of its domain plastic deformations or plastic regime under the boundary conditions and the defined load scenario (Figure 21 and Figure 22) for both geometries. On the other hand, Figure 27 and Figure 28 show the displacement field along the BIM construction elements under study. As can be verified, the maximum displacements were located in geometrical areas of the construction elements that have the greatest distance between the different supports. However, the magnitude of these maximum displacements was 0.267 mm (E_1_, Figure 27) and 0.545 mm (E_2_, Figure 28), a magnitude that could be defined as admissible if we considered the spatial scale of the constructive BIM elements and also that these displacements did not pose a conflict for the structural integrity of the BIM construction elements.

### 2.3. Definition of the Construction Elements in a BIM Environment

**Figure 29:**
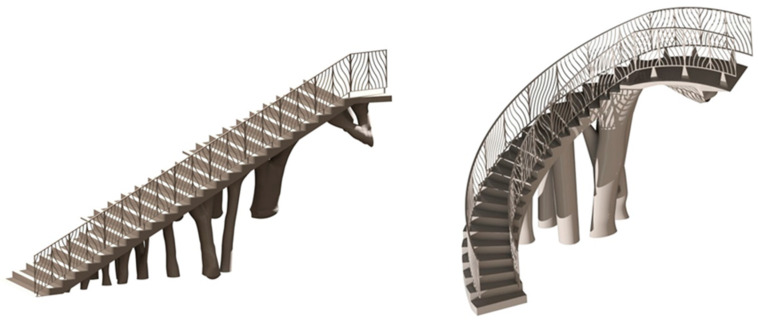
Geometrical models of the construction elements under study, imported into the BIM environment E_1_ (**left**) and E_2_ (**right**).

Starting from the geometrical modeling of the construction elements (Figure 20) and after numerically evaluating their structural integrity, both plastic geometries were implemented in a BIM environment. The commercial software used for the management of data and attributes of both structural elements was AECOSim Building Designer v8i [57]. The methodology begins by importing the constructive geometry from the CAD software CATIA V5-R21 [54] where the geometry was modeled, using a neutral geometrical exporting procedure via STEP format. These geometrical files allow transferring geometrical information between both softwares, as well as importing the final topologies in AECOSim Building Designer v8i. Figure 29 shows a rendered model of the results of importing the geometrical elements into the BIM environment.

The methodology for implementing an element in a BIM environment requires the definition and organized designation of a set of properties and characteristics in subgroups, according to its geometrical, aesthetical and technological parameters. This set of properties and characteristics is defined as catalog or Datagroup (nomenclature established by the commercial software), having the possibility of being qualitative or quantitative. In this way, each constructive element presents its own and individualized catalog definition and designation, despite the fact that they can share similar properties and characteristics among various BIM elements. Table 10 shows the groups of properties and features used to define the catalogs of the BIM construction elements under study in this manuscript.

However, since the manufacturing of the construction BIM elements which are the object of our study was carried out by means of 3D fused deposition modeling (FDM) and plastic materials, it was required to define a new group (specified in Table 10 as manufacturing) associated with plastic manufacturing to be applied in catalogs or Datagroups. This group must include the different properties and technological features associated with FDM additive manufacturing technology. The generation of this new 3D FDM technology group is required, since commercial software with a BIM environment only presents the groups associated with conventional manufacturing processes and traditional construction methods. Table 11 shows the new properties and technological features defined in this manuscript for the BIM environments and 3D additive manufacturing that have been applied to the BIM construction elements under study. In this way, from this new group (see Table 11), any element whose manufacturing process is 3D FDM technology with polymeric materials can be implemented in a BIM environment.

In order to complete the implementation of the construction elements in the BIM environment, a new set of geometrical and technological attributes, related to the measurements and unit prices involved in calculating the total manufacturing cost of the elements under study were determined. These attributes, those associated with both geometry and technology, make up the Dataset (nomenclature established by the commercial software used) or family for each construction element. The attributes defined are: volume, total area, lateral area, manufacturing unit price, plastic material density and mass. The total manufacturing cost of each constructive element in the BIM environment is obtained from an analytical model (Equation (9)) [57] made up of three concepts which intervene in the 3D FDM technology: operation cost, material cost and labor cost:

(9) C=O+M+W

where, C (€) represents the total manufacturing cost, O (€) represents the cost of the additive printing operation using the FDM process, M (€) represents the cost associated with the material used during manufacturing and W (€) represents the cost of labor. Furthermore, each concept established in Equation (9) can be defined analytically as shown in Equations (10)–(12):

(10) $$O=t_{o}\cdot C_{o}\;$$

(11) $$M=V_{m}\cdot \rho_{m}\cdot C_{m}\;$$

(12) $$W=t_{w}\cdot C_{w}=0.25\cdot t_{o}\cdot C_{w}\;$$

where t_o_ (s) represents the total manufacturing time of the constructive element, C_o_ (€/s) represents the unit cost associated with the 3D additive manufacturing operation using the FDM technology, V_m_ (m^3^) represents the total volume of polymeric material used during the manufacturing process, ρ_m_ (kg/m^3^) represents the density of the polymeric material, C_m_ (€/kg) represents the unit cost of the plastic material, t_w_ (s) represents the total time used by the operators during the manufacturing process and C_w_ (€/s) represents the unit cost of the operators involved in the manufacturing process. As shown in Equation (12), the total time used by the operators during the manufacturing process t_w_ can be assumed as a quarter of the total manufacturing time of the construction element [58].

**Figure 30:**
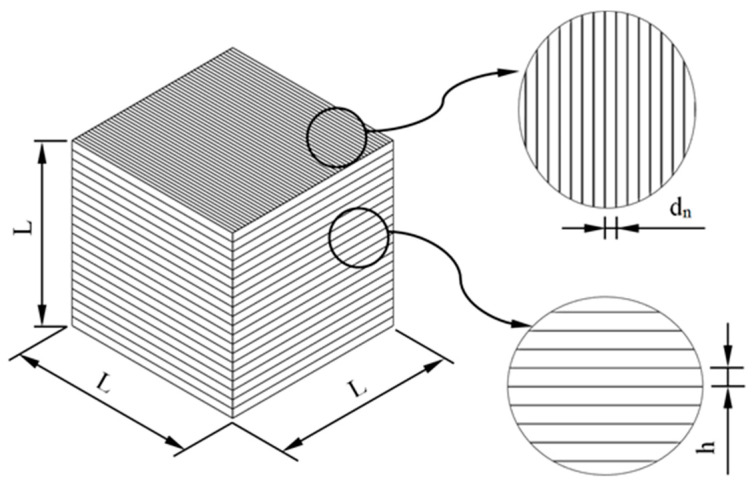
Representation of the cubic element parameterized according to the 3D printing technological parameters layer height h (m) and diameter of the nozzle d_n_ (m).

In order to carry out the analytical process of calculating the total manufacturing cost of each construction element in an automated way for the BIM environment, the paper proposes a new algorithm based on the discretization of the continuous geometry of structural elements in 3D voxels. Applying this methodology to a discrete cubic volume, the manufacturing time t_oc_ can be determined according to Equations (13) and (14):

(13) $$t_{\mathrm{oc}}=\frac{L_{i}}{v_{i}}\;$$

(14) $$L_{i}=n_{\mathrm{layers}}\cdot L_{\mathrm{layers}}\;$$

where L_i_ (m) represents the distance traveled by the extruder to complete the manufacture of a cubic geometry and v_i_ (m/s) represents the speed of the extruder along this path. As shown in Figure 30, the distance traveled by the extruder to complete the manufacture of a cubic geometry L_i_, can be defined as the number of vertical layers used in its manufacture n_layers_ by the length that the extruder travels during each vertical layer L_layers_ (Equation (14)).

As shown in Figure 30, the number of vertical layers n_layers_ can be defined as the relationship between the dimension of the cube L and the technological parameter layer height h. The distance traveled by the extruder to manufacture each vertical layer L_layers_ can be defined as the relationship between the area of the base of the cubic element L^2^ (Figure 30) and the technological parameter corresponding to the diameter of the extruder d_n_. In this way, the definition of the total time for manufacturing a cubic geometry (Equation (15)) was established from the 3D additive manufacturing technological parameters: layer height, nozzle diameter and extruder speed (Equation (16)):

(15) $$t_{\mathrm{oc}}=\frac{L_{i}}{v_{i}}=n_{\mathrm{layers}}\cdot L_{\mathrm{layers}}\cdot \frac{1}{v_{i}}=\frac{L}{h}\cdot \frac{L^{2}}{d_{n}}\cdot \frac{1}{v_{i}}=\frac{L^{3}}{h\cdot d_{n}\cdot v_{i}}\;$$

(16) $$t_{\mathrm{oc}}:=t_{\mathrm{oc}}\left(h,{\;d}_{n},{\;v}_{i}\right)\;$$

**Figure 31:**
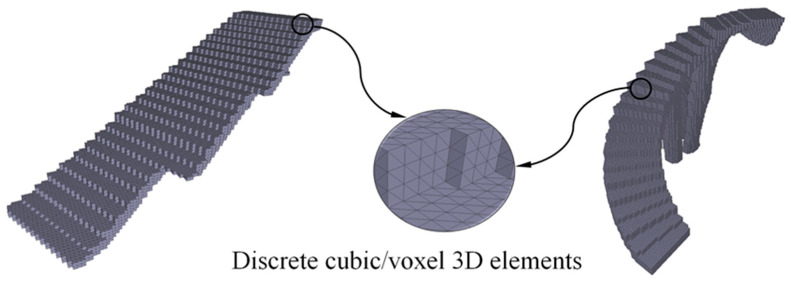
Discretization of the geometry of the constructive elements using cubes or 3D voxels.

Then, once the time for manufacturing a discrete cubic element has been defined, and since the constructive elements are discretized according to cubic geometries, the analytical total manufacturing time of the constructive elements is established by a superposition process (Equation (17)). That is, the manufacturing time is determined as the product of the number of 3D cubes or voxels (Figure 31), which discretizes the geometry of the construction elements, and the discrete manufacturing time for a cubic geometry:

(17) $$t_{o}≅t_{\mathrm{oc}}\cdot n_{c}≅\frac{L^{3}\cdot n_{c}}{h\cdot d_{n}\cdot v_{i}}\;$$

where n_c_ represents the number of cubes or 3D voxel (Figure 31) that discretizes the geometry of the construction elements. The main dimension for the cubic element or voxel 3D L was determined according to the dimensions of the minimum detail of each constructive element studied in this manuscript. In a similar way, substituting Equation (16) for Equations (10), (12) and (9), it was possible to propose an analytical equation for obtaining the total manufacturing cost of each item manufactured with 3D printing (Equation (18)):

(18) $$\begin{array}{ll} C=O+M+W & =t_{o}\cdot C_{o}+V_{m}\cdot \rho_{m}\cdot C_{m}+0.25\cdot t_{o}\cdot C_{w}\\ & =t_{o}\cdot \left(C_{o}+0.25\cdot C_{w}\right)+V_{m}\cdot \rho_{m}\cdot C_{m}=\\ & =\frac{n_{c}\cdot L^{3}}{h\cdot d_{n}\cdot v_{i}}\cdot \left(C_{o}+0.25\cdot C_{w}\right)+V_{m}\cdot \rho_{m}\cdot C_{m}\; \end{array}$$

After establishing the new algorithm that determined the total manufacturing cost for an element implemented in the BIM environment and whose manufacturing process was FDM 3D printing, Table 12 shows the set of geometrical and technological attributes that define the family or Dataset of the constructive element under study. As shown, Table 12 classifies two main groups of attributes. Firstly, the geometrical attributes that are automatically defined by the measurements made by commercial software on the construction elements and secondly, the new technological attributes that must be defined by the user according to the type of FDM 3D printer, the geometry of the element implemented in the BIM environment, the thermoplastic material and the unit costs defined by the industrial company. In this way, it is possible to link the geometry of the structural element with the technological, functional and economic information necessary to work in advanced construction 4.0 environments, obtaining an expanded three-dimensional model.

To complete the implementation of the construction elements in the BIM environment, it can be established that both construction elements under study (Figure 20 belongs to the same family or Dataset, given that both the geometrical and technological attributes associated with the manufacturing process, as well as the methodology for calculating the total manufacturing cost, are analogous.

**Figure 32:**
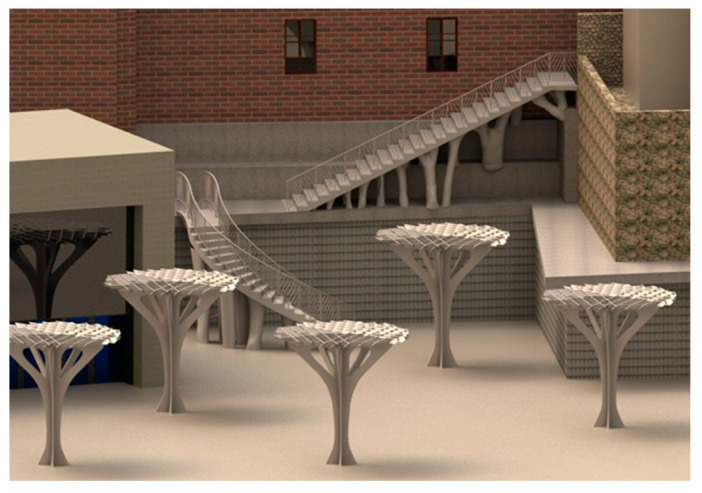
Digitally rendered model of the implementation of BIM construction elements in the urban environment.

Finally, and given that the urban environment was digitized, a final visualization of the implementation results of the expanded three-dimensional models for the construction elements under study is presented in Figure 32. Additionally, Figure 32 shows the complete models where the functionality and accessibility provided by the construction elements developed in this manuscript is evaluated. It has been possible to achieve an architectural environment with high impact and originality manufactured with polymeric materials improving the urban environment where the developed structural elements will be located.

## 3. Conclusions

The work developed in this manuscript presents a new procedure for the BIM structural objects manufactured using FDM additive technology and polymeric materials. This new procedure transforms the geometry of the architectural structure into a three-dimensional expanded digital model capable of directly linking the topological information of the structural element with the technological, functional and economic information needed for working in advanced construction 4.0 environments. The model presented incorporates an innovative geometrical algorithm whose objective is to obtain, in a fully automated way, the FDM manufacturing time and the manufacturing cost for BIM plastic structural objects. The new algorithm starts from the voxelized geometrical surface of the architectural model, calculating the manufacturing time from the full geometric path traveled by the extruder in a voxel, the extruder speed, the print pattern and the layer height. The voxel size is determined by the minimum geometric detail of the structural BIM element. 

Two innovative free-form structural BIM objects were designed, taking advantage of using PETG plastic material and FDM manufacturing technology. These architectural elements were designed with free form geometries according to the aesthetic, functional and technical requirements of the urbanistic implantation. The digital architectural environment was obtained by the means of photogrammetric reverse engineering technologies. The final geometry of the BIM architectural elements was obtained by applying topological optimization processes from the scenario of loads and boundary conditions required by the regulations. In this way, it has been possible to reduce the amount of plastic material, resulting in a strong, sustainable and highly visual architectural geometry. The BIM architectural elements have been validated from the structural point of view by numerical simulation, following the scenario of loads and boundary conditions used in the topological optimization process. The von Mises stress field presents maximum values of 0.423 MPa and 0.650 MPa, for both plastic structural objects without exceeding in any case the tensile yield strength of the thermoplastic material. The resulting displacement map shows maximum values of 0.237 mm and 0.5 mm, respectively, in the two case studies, these being admissible magnitudes considering the spatial scale of the cases studied.

A new expanded digital model applicable to BIM objects associated with 3D FDM additive manufacturing was developed. This new model includes the corresponding properties and technological features associated with the FDM manufacturing process and polymeric materials, allowing the definition of any element in an advanced digital BIM environment whose manufacturing process is as indicated. In this way, it is possible to obtain for structural elements made with polymeric materials and FDM technology a complete digital model, capable of incorporating, automating and managing in real time the information and calculations necessary for manufacturing, validating and analyzing the plastic architectural object in advanced BIM environments. The research presented in this paper is a comprehensive proposal on the industrial and research level in the field of construction 4.0 and it enhances the advantages of using polymers in additive FDM manufacturing environments. Therefore, professionals wanting to formulate new polymeric materials and use FDM technology with polymers in BIM construction 4.0 environments can take advantage of using the work methodology presented in this paper.

The results presented suggest that the use of FDM technology along with PETG material is promising for the manufacture of architectural components that are subject to compression efforts. The extended BIM model developed by the authors is applicable in any BIM environment and for new polymeric materials with independence of the structural element digital model format.

## Figures and Tables

**Table 1:** Statistical results of data collection obtained after the aerotriangulation process

Goodness of Fit	Values
Number of points	12.171,000
Median reprojection error	0,170
RMS of reprojection errors	0,350
RMS of distances to rays	0,003

**Table 2:** Technical specifications of the OLIMPUS E-500 DSLR digital camera and the taken photographs.

Technical Specifications	Units	Properties
Dimensions	mm	3264 × 2448
Resolution	px	314 × 314
Lens model	-	Olumpus Zuiko Digital: 14–80 mm F3.5–5.6
Measuring range of the tilt sensor	-	
Shutter speed	s	1/80
Focal length	mm	30
ISO speed	-	125

**Table 3:** Technical specifications of the Leica Disto D510 (E7500i) digital laser distance meter.

Technical Specifications	Units	Values
Measurement accuracy	mm	1
Scope	m	200
Distance in m for laser spot in mm	m/mm	10, 50, 100/6, 30, 60
Measuring range of the tilt sensor	°	360
Dimensions	mm	143 × 58 × 29
Height	g	198

**Table 4:** Maximum dimensions of the bounding boxes B_1_ and B_2._

l_1_ (m)	w_1_ (m)	h_1_ (m)	l_2_ (m)	w_2_ (m)	h_2_ (m)
13.082	3.949	3.756	6.111	4.039	4.011

**Table 5:** Physical, mechanical and technological properties of polyethylene terephthalate glycol (PETG).

Variable	Units	Values
Melting range	°C	210–280
Density	g/cm^3^	1.27
Flexural Modulus	MPa	2100
Flexural Strength	MPa	69
Notched Izod Impact	J/m	105
Rockwell Hardness	R scale	108R
Tensile Yield Strength	MPa	50
Poisson coefficient	-	0.38
Heat Distortion Temperature	°C	70–80
Vicat Softening Temperature	°C	85
Print Temperature	°C	235 ± 10
Hot Pad	°C	60–90

**Table 6:** Statistics of the discrete mesh associated with the bounding boxes B_1_ and B_2_.

Variable	Unit	B_1_, Value	B_2_, Value
Number of elements	-	224,234	45,920
Number of nodes	-	320,531	68,433
Average aspect ratio	-	1.590	2.430
Average shape factor	-	1.846	0.667
Average max edge length	mm	147.000	170.700
Average min edge length	mm	93.500	106.400

**Table 7:** Statistics of the volume resulting from the topological optimization algorithm for the bounding boxes B_1_ and B_2._

Variable	Unit	B_1_, Value	B_2_, Value
Bounding box volume	m^3^	143.058	11.859
Optimized geometry volume	m^3^	6.558	3.288
Volume reduction percentage	%	95.416	72.274

**Table 8:** Statistics of the discrete mesh associated with the BIM E_1_ and E_2_ constructive elements.

Variable	Unit	B_1_, Value	B_2_, Value
Number of elements	-	310,345	384,933
Number of nodes	-	65,295	560,643
Average aspect ratio	-	1.600	1.590
Average shape factor	-	0.683	0.683
Average max edge length	mm	74.980	63.100
Average min edge length	mm	47.350	39.930

**Table 9:** Results of the numerical mechanical analyses of the BIM, E_1_ and E_2_ construction elements.

BIM Type Building Element	Numerical max. von Mises Stress (MPa)	Numerical max. Displacement (mm)
E_1_	0.423	0.267
E_2_	0.650	0.545

**Table 10:** Group set used to define the catalog or Datagroup of the constructive BIM elements E_1_ and E_2_.

BIM Datagroups Definition
Accessibility
Discipline
Manufacturer
Identity
Phasing
Thermal transmittance
Application
Common
Quantities
Classification
IFC over-ride
Manufacturing

**Table 11:** Properties and technological characteristics used in the definition of the group associated with the manufacture of the BIM, the E_1_ and E_2_ construction elements.

Group of Properties	Property Definition	Variable Type
Quality	Layer height	Decimal
	Material	Chain
	Nozzle	List
Shell	Bottom layers	Decimal
	Bottom thickness	Decimal
	Horizontal expansion	Decimal
	Top layer	Decimal
	Top thickness	Decimal
	Wall line count	Decimal
In-fill	Wall thickness	Decimal
	In-fill density	Decimal
	In-fill pattern	List
Material retraction	Materialretract	Boolean
Speed	Print speed	Decimal
Travel	Zhop when retracted	Boolean
Cooling	Enable print cooling	Boolean
	Fan speed	Decimal
Support	Build plate adhesion	List
	Generate support	Boolean
	Support overhang angle	Decimal
	Support placement	List

**Table 12:** Definition of the properties and the technological characteristics used in the definition of the family or Dataset of the BIM E_1_ and E_2_ construction elements.

**Technological Attributes of the Dataset, Defined by the User**		
**Variable**	**Units**	**Nomenclature**
Dimension of the cube	m	L
Number of cubes for geometric discretization	-	n_c_
Layer height	m	h
Extruder diameter	m	D_n_
Extruder speed	m/s	V_i_
Density of the material	kg/m^3^	ρ_m_
Unit cost of operation	€/s	C_o_
Unit cost of the material	€/kg	C_m_
Unit cost of labor	€/s	C_w_
**Dataset Geometric Attributes, Measurements**		
**Variable**	**Units**	**Nomenclature**
Volume	m^3^	V_m_
